# Recombinant Human Thyroid-Stimulating Hormone Increases the Percentages of Natural Killer T Cells and B Lymphocytes in Human Peripheral Blood *In Vivo*

**DOI:** 10.3389/fendo.2020.543845

**Published:** 2020-11-20

**Authors:** Zbigniew Adamczewski, Mariusz Stasiołek, Arkadiusz Zygmunt, Przemysław W. Śliwka, Katarzyna Wieczorek-Szukała, Andrzej Lewiński

**Affiliations:** ^1^ Department of Endocrinology and Metabolic Diseases, Medical University of Lodz, Lodz, Poland; ^2^ Department of Neurology, Medical University of Lodz, Lodz, Poland

**Keywords:** thyroid-stimulating hormone (TSH), dendritic cells, monocytes, thyroid, lymphocytes, natural killer (NK) cells, NKT cells

## Abstract

Multiple cellular and humoral components of the immune system play a significant role in the physiology and pathophysiology of various organs including the thyroid. On the other hand, both thyroid hormones and thyroid-stimulating hormone (TSH) have been shown to exert immunoregulatory activities, which are difficult to assess independently *in vivo*. In our study we employed a unique clinical model for the assessment of TSH biological function in humans. The structure of peripheral blood mononuclear cell populations was investigated, using flow cytometry, in athyroid patients (n = 109) after treatment because of the differentiated thyroid carcinoma (DTC) at two time-points: directly before and five days after recombinant human TSH (rhTSH) administration. The analysis revealed significant increase in the percentage of natural killer T cells and B lymphocytes in the peripheral blood of rhTSH treated patients, whereas, we did not observe any effects on investigated subpopulations of dendritic cells and monocytes, T cells and natural killer cells. The findings of the study indicate the immune regulatory role of TSH, directed specifically on selected cell subtypes.

## Introduction

The immune and endocrine systems remain functionally connected and the relations between numerous cell types and humoral mediators are complex and not yet fully understood (1). The influence of the immune system on thyroid function has been investigated in various animal experimental models as well as in human thyroid disorders (2, 3). Accumulating evidence points at dendritic cells (DCs) as crucial players in the regulation of inflammatory events associated with thyroid pathology [reviewed in (4)]. DCs are considered as the most potent antigen presenting cells with strong ability to acquire and process exogenous and endogenous antigens and present them to naïve T lymphocytes. The broad expression of co-stimulatory molecules and secretion profile allows DCs to direct the differentiation and activation processes of these lymphocytes. DCs are involved not only in the activation of naïve T lymphocytes but also in a variety of cross-interactions with other immune cells. The extraordinary regulatory properties of DCs depend strongly on their maturity and activation state and differ considerably between particular DC subpopulations. In human settings, two main subpopulations of DCs—conventional/myeloid and plasmacytoid DCs (cDCs and pDCs, respectively) are commonly recognized (5–7). It is of great importance that the immune system and thyroid interactions may be also mediated by thyroid hormones (THs). The expression of thyroid hormone receptor (TR) was described in various immune cell types *e.g.* monocytes, macrophages, natural killer cells (NK cells), lymphocytes and DCs (8). An analysis of molecular mechanisms underlying the action of THs on murine bone marrow derived DCs suggested an involvement of signal transduction pathways associated with Akt and the NF-*κ*B transcription factors (Nuclear Factor kappa-light-chain-enhancer of activated B cells) (9). Not only the direct influence of THs on the elements of the immune system, but also aberrations in the hypothalamic–pituitary–thyroid axis function including changes of thyrotropin (Thyroid-Stimulating Hormone, TSH) levels may be of significance. TSH receptors (TSHR) are present on the surface of B and T lymphocytes, NK cells, monocytes, and are at high levels in DCs (8). It was shown that TSH increased the phagocytic activity of murine DCs and selectively enhanced the secretion of interleukin (IL)-1β and IL-12 by DCs stimulated with an inducer of phagocytic activity (10). In combination with IL-2, TSH exerted also costimulatory activity on murine NK cells (11).

The high expression of TSHR on murine DCs may suggest the involvement of those cells in the immunoregulatory processes associated with the changes of thyrometabolic state. The majority of TSHR + DCs were found to exhibit myeloid subpopulation characteristics based on the expression of MHC class II and co-expression of CD11c and CD11b molecules (10). However, due to the complexity of thyrometabolic state regulation and methodological difficulties, very little is known about the effects exerted by TH and TSH on human immune system.

In our study we employed a unique clinical model for the assessment of TSH biological function in humans independently of TH influence. The study was performed in patients after therapy (total thyroidectomy or total thyroidectomy and ^131^I radioiodine ablation therapy) because of differentiated thyroid carcinoma (DTC) and qualified for recombinant human TSH (rhTSH) administration from standard indications. In our previous, preliminary experiments using this clinical model, we showed that the systemic administration of rhTSH did not influence the quantitative or phenotypic parameters of the peripheral blood DC subpopulations (12). In the current study we extended both the number of the patients and the spectrum of the analysis in order to assess the influence of TSH on multiple immune cell populations (including peripheral blood monocyte and DC subpopulations, B lymphocytes, T lymphocytes, NKT cells, and NK cell subpopulations) in humans under *in vivo* conditions allowing for the exclusion of effects of TH level fluctuations.

## Materials and Methods

### Patients

The study participants were recruited from the Department of Endocrinology and Metabolic Diseases, Polish Mother’s Memorial Hospital—Research Institute in Lodz. Prior to the enrolment, all of the participants signed an informed consent according to the study protocol approved by the local Ethics Committee. The study group included 109 patients (97 women and 12 men, age 51.7 ± 12.8 years, mean ± SD) after treatment because of the DTC (total thyroidectomy—n = 23, total thyroidectomy and ^131^I radioiodine ablation therapy—n = 86).

The patients with persisting active neoplastic disease, immunological or metabolic disorders, as well as patients with clinical or laboratory signs of ongoing inflammatory processes were excluded from the study. Recombinant human TSH (rhTSH, Thyrogen, Sanofi Genzyme) was administered according to a standard regimen as an intramuscular injection (two injections on consecutive days, 0.9 mg each) as a routine control of potential thyroid cancer activity. Peripheral blood samples were collected between 08.00 and 09.00 AM after an overnight fast. Venous blood was obtained by clean venipuncture (needle gauge 19), avoiding slow flowing draws and/or traumatic venipunctures. The blood samples were collected from the same patient (n = 109) at two (2) consecutive time points: (i) directly before the commencement of rhTSH administration and (ii) five (5) days after first rhTSH injection.

Free triiodothyronine (FT3), free thyroxine (FT4), and TSH concentrations were measured by the immunoradiometric (IRMA) method with appropriate laboratory kits (Roche Diagnostic Mannheim, Germany; range normal values: TSH: 0.27–4.2 mIU/L; FT3: 2.6–4.4 pg/ml; FT4: 0.93–1.7 ng/dl).

### Fluorescence-Activated Cell Sorting Analysis

Whole blood samples obtained from the study participants were assessed on the same day by flow cytometry, using a FACSCanto II cytometer and FACSDiva software (BD Biosciences, San Jose, CA, USA). The study encompassed the prospective analysis of multiple peripheral blood immune parameters in the same patients (n = 109) before and 5 days after systemic rhTSH administration. The interim analysis (n = 46) revealed significant changes in the non-monocytic CD16 positive cell fraction, indicating the influence of rhTSH on NK cells. Because of that observation additional parameters, enabling an assessment of NK and NKT cells, were introduced in the further measurements (n = 63).

The peripheral blood samples were subjected to extracellular staining with following fluorochrome-conjugated monoclonal antibodies (mAbs):

- fluorescein isothiocyanate (FITC) conjugated mAb: anti-CD16 (3G8, Mouse IgG1) (BD Biosciences Pharmingen, San Jose, CA, USA),
- phycoerythrin (PE) conjugated mAb: anti-CD86 (2331 (FUN-1), Mouse IgG1) (BD Biosciences), anti-CD11c (B-ly6, Mouse IgG1) (BD Biosciences),
- peridinin–chlorophyll–protein complex (PerCP) conjugated mAb: anti-CD14 (M5E2, Mouse IgG2a) (BD Biosciences), anti-CD19 (4G7, Mouse IgG1) (BD Biosciences),
- allophycocyanin (APC) conjugated mAb: anti-CD3 (UCHT1, Mouse IgG1) (BD Biosciences), anti-BDCA1-(CD1c) (AD5-8E7, Mouse IgG2a) (Miltenyi Biotec), anti-BDCA2-(CD303) (AC144, Mouse IgG1) (Miltenyi Biotec), anti-BDCA3-(CD141) (AD5-14H12, Mouse IgG1) (Miltenyi Biotec),
- phycoerythrin–cyanin conjugate 7 (PE-Cy7) conjugated mAb: anti-CD56 (B159, Mouse IgG1) (BD Biosciences).

Post-staining red blood cell lysis was performed using BD FACS lysing solution (BD Biosciences Pharmingen, San Jose, CA, USA). Before measurement, samples were washed in PBS and centrifuged at 1,400 RPM for 5 min at 20°C. To avoid an unspecific antibody-binding, a FcR blocking reagent (Miltenyi Biotec, Bergisch Gladbach, Germany) was applied in all analyses.

Individual populations of immune cells were identified by their expression of surface antigens:

- DC subsets were recognized on the basis of expression of a panel of surface molecules known as Blood Dendritic Cell Antigens (BDCAs) and CD19
- pDCs—BDCA2 (CD303)^+^
- cDCs1—BDCA1 (CD1c)^+^ CD19^−^
- cDCs2—BDCA3 (CD141)^high^
- Monocyte subsets were recognized on the basis of the surface expression level of CD14 and CD16 molecules
- T lymphocytes—CD3^+^
- B Lymphocyte—CD19^+^
- NK cells—CD3^−^CD56^+^ cells—subpopulations of NK cells were recognized on the basis of the surface expression level of CD16 molecule
- NKT cells—CD3^+^CD56^+^ cells

At least 20,000 cells were counted in the mononuclear leukocyte gate in each sample.

### Statistical Analysis

The statistical analysis was carried out using the Statistica 12 software (TIBCO Software, Palo Alto, CA, USA). A graphical representation of the results was prepared using the SigmaPlot 11 software (Systat Software Inc., San Jose, CA, USA). The normality of distribution was assessed utilizing the Shaphiro–Wilk test, and the differences in peripheral blood cell populations were analyzed with the Wilcoxon matched-pairs signed rank test. In all the analyses, results were considered statistically significant when p < 0.05.

## Results

### Clinical Characteristics

All the study participants received L-T4 treatment in individually adjusted doses (mean L-T4 dose ± SD: 138.6 ± 35.0 μg/day). Mean serum concentrations of chosen parameters at the commencement of the study—before first rhTSH injection (day 1) and at the end of the study (day 5) are shown in **Table 1**.

**Table 1:** Serum concentration of chosen parameters.

Parameter	Day 1(mean ± SD)	Day 5(mean ± SD)	Reference ranges	P value
Free triiodothyronine (FT3) (pg/ml)	3.02 ± 0.54	3.14 ± 0.60	2.6–4.4	0.28
Free thyroxine (FT4) (ng/dl)	1.65 ± 0.33	1.82 ± 0.44	0.93–1.7	0.016
Thyroid-stimulating hormone (TSH) (mIU/L)	0.49 ± 0.90	31.12 ± 18.44	0.27–4.2	<0.001

### rhTSH Impact on T and B Lymphocytes

**Figure 1:**
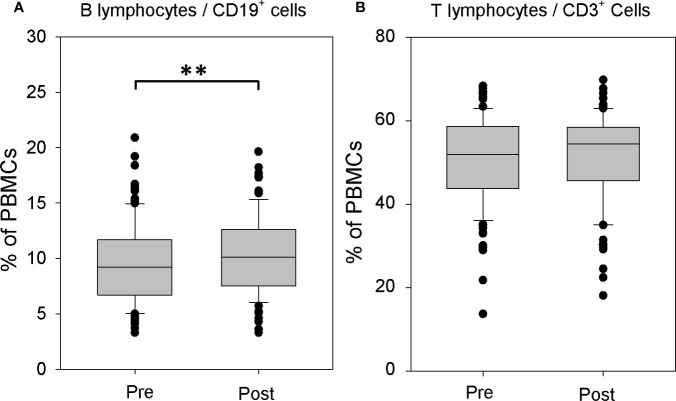
The influence of recombinant human TSH (rhTSH) on distribution of human T and B lymphocytes. The percentage of B lymphocytes **(A)** and T lymphocytes **(B)** in the whole peripheral blood mononuclear cells (PBMC) fraction of patients (n = 109) directly before (pre) and on the fifth day of the study (post) (**p < 0.01).

The comparison of the structure of immune cell populations before and after systemic rhTSH administration in athyroid DTC patients revealed significant increase of the percentage of CD19^+^ B lymphocytes in the peripheral blood mononuclear cells (PBMCs) after rhTSH treatment (9.60 ± 4.09 *vs*. 10.29 ± 3.52%, p = 0.003), whereas, the percentage of CD3^+^ T lymphocytes remained unaffected by rhTSH administration (50.54 ± 10.45 *vs*. 51.52 ± 10.46%) (**Figure 1**).

### rhTSH Impact on CD16+ Expressing Cells

The percentage of PBMCs expressing CD16 did not change after rhTSH administration (13.08 ± 6.52 *vs*. 13.83 ± 6.17%). The lack of rhTSH effect was observed both in monocytic CD14^+^CD16^+^ (2.94 ± 2.82 *vs*. 3.10 ± 2.55%) as well as non-monocytic CD14^−^CD16^+^ fraction (10.34 ± 5.68 *vs*. 10.63 ± 5.69%) (**Supplementary Figure 1**).

### rhTSH Impact on Monocytes

**Figure 2:**
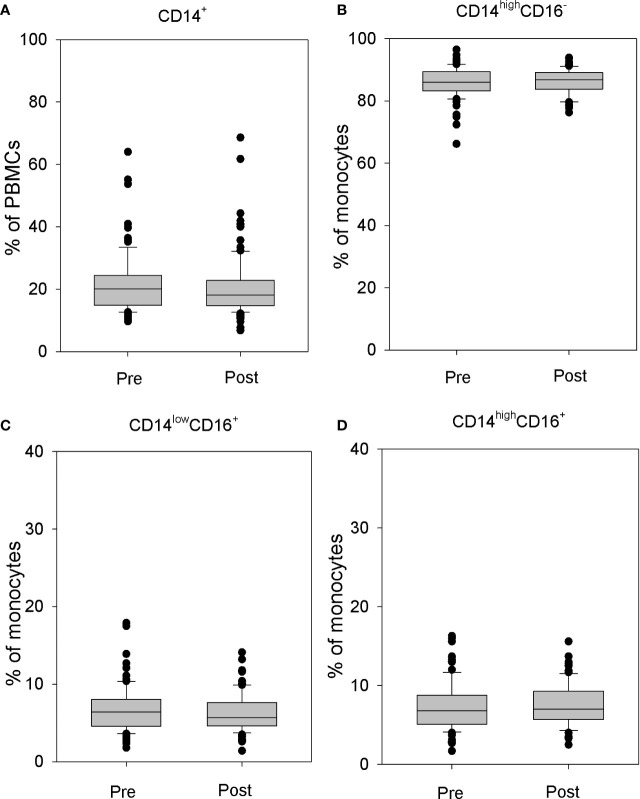
The influence of rhTSH on distribution of human CD14^+^ cells. The percentage of CD14^+^ cells in the whole PBMC fraction **(A)**, the percentage of CD14^high^CD16^-^ classical **(B)**, CD14^low^CD16^+^ non-classical **(C)** and CD14^high^CD16^+^ intermediate **(D)** monocytes in the monocyte fraction of patients (n = 109) directly before (pre) and on the fifth day of the study (post).

Detailed analysis of monocyte fraction showed no effect of rhTSH administration either on the whole peripheral blood monocyte population (**Figure 2A**) or on monocyte subpopulations defined on the basis of the CD16 and CD14 expression (**Figures 2B–D**).

Monocytes were divided into three subpopulations: CD14^high^CD16^−^ classical monocytes, CD14^low^CD16^+^ non-classical monocytes, and CD14^high^CD16^+^ intermediate monocytes (13). No changes in the percentage of classical monocytes (86.25 ± 4.39 *vs.* 86.18 ± 3.98%) (**Figure 2B**), non-classical monocytes (6.40 ± 2.62 *vs*. 6.23 ± 2.34%) (**Figure 2C**), or intermediate monocytes (7.33 ± 2.90 *vs*. 7.58 ± 2.57%) (**Figure 2D**) were observed.

### rhTSH Impact on NK Cells and NKT Cells

Because of the results of the interim analysis (n = 46), which indicated an existence of significant changes in the non-monocytic CD16 positive cell fraction, additional parameters, enabling an assessment of NK and NKT cells, were introduced in the further measurements in 63 patients.

**Figure 3:**
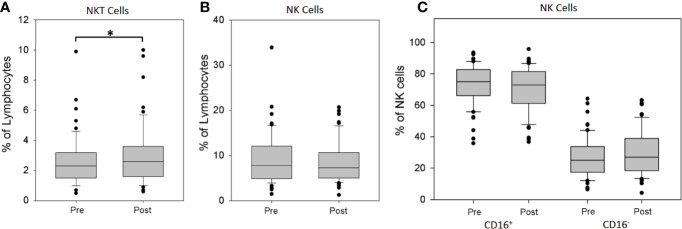
The influence of rhTSH on distribution of human NK and NKT cells. The percentage of NKT cells **(A)**, NK cells **(B)** and NK cells subsets **(C)** in the lymphocyte fraction of patients (n = 63) directly before (pre) and on the fifth day of the study (post) (*p < 0.05).

The sequential analysis of peripheral blood showed a significant increase of the percentage of NKT cells in the lymphocyte fraction after rhTSH administration (2.67 ± 1.73 *vs.* 3.14 ± 2.17%, p = 0.015) (**Figure 3A**). To the contrary, there was no change of the percentage of NK cells (8.58 ± 4.54 *vs*. 8.54 ± 4.40%) (**Figure 3B**). Further analysis of NK cell subpopulations showed that rhTSH administration did not change the percentage of either CD16^+^ (73.27 ± 12.74 *vs*. 70.44 ± 14.16%) or CD16^−^ NK cells (26.73 ± 12.74 *vs* 29.56 ± 14.16%) (**Figure 3C**).

### rhTSH Impact on Dendritic Cells

**Figure 4:**
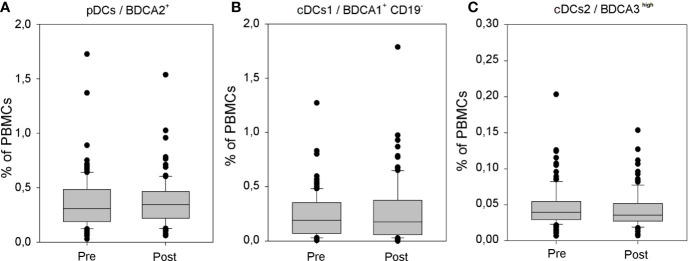
The influence of rhTSH on distribution of human peripheral blood dendritic cell (DC) subpopulations. The percentage of plasmacytoid DCs (pDCs) **(A)**, CD1c/BDCA1^+^ CD19^−^ conventional/myeloid DCs (cDCs) **(B)** and CD141/BDCA3^high^ cDCs **(C)** in the whole PBMC fraction of patients (n = 109) directly before (pre) and on the fifth day of the study (post).

In accordance with the results of our preliminary analysis (12), also the assessment performed on the significantly larger group of athyroid DTC patients did not reveal any effects of rhTSH administration on the percentage of peripheral blood conventional (0.24 ± 0.21 *vs*. 0.27 ± 0.25%) and plasmacytoid (0.36 ± 0.25 *vs*. 0.39 ± 0.30%) DC populations. Importantly, the percentage of CD141/BDCA3^high^ cDCs was very low in all patients and did not show any significant quantitative fluctuations during the study (0.046 ± 0.028 *vs*. 0.042 ± 0.025) (**Figure 4**). Administration of rhTSH had also no effect on the level of surface expression of one of the major costimulatory molecules—CD86 in particular DC populations (**Supplementary Figure 2**).

## Discussion

The direct effects of TSH are mediated by TSHR, transmembrane receptor highly expressed by thyrocytes. The expression of this receptor can be also detected in the cells of various tissue types including the cells of the immune system. Despite the low TSHR expression outside the thyroid, the very high binding affinity for TSH allows the hormone to activate a cellular response even in the case of low density of TSHR on cell surface (14).

The functional meaning of TSH outside the thyroid gland is not fully determined. It has been suggested that TSH may act as a modulator, operating at multiple different systems and organs (15–24). Under clinical conditions similar to the ones applied in our study, rhTSH was shown to influence serum ghrelin levels (19), as well as serum levels of total cholesterol and triglycerides without any significant changes of high and low lipoproteins (20). Another study demonstrated that the serum level of platelet microparticles in athyroid patients rose fivefold after rhTSH stimulation (21). Exogenous rhTSH was also shown to have an impact on the cardiovascular system, where systolic and diastolic blood pressure increased 5 days after administration (22). What is more, rhTSH administration was associated with significant deterioration of renal perfusion and reduction in glomerular filtration (23). On the other hand, the protective effect of rhTSH on the coronary endothelium, resulting in an increase of coronary blood flow was observed in a small group of athyroid patients undergoing echo-Doppler examination with evaluation of the coronary flow reserve (24).

The effects exerted by TSH on immune cells are not sufficiently described, and the available data were obtained mainly with the use of animal models or in *in vitro* assays with human PBMCs. TSHR expression was detected in murine DCs and lymphocytes. Interestingly the level of TSHR expression differed substantially between particular lymphoid organs. Approximately 2–3% of murine splenic CD4^+^ or CD8^+^ T lymphocytes and CD19^+^ B lymphocytes and 10–20% of lymph node lymphocytes were shown to express TSHR. The receptor density was very low in splenic T lymphocytes, whereas, in lymph nodes significant proportion of T lymphocytes expressed TSHR at high level (10). In human PBMCs, binding of biotinylated TSH was shown mainly to monocytes and NK cells (25). In animal models, TSH significantly stimulated secretory and phagocytic activity of DCs (8, 10). Animal experiments indicated also that at least some of the immunomodulatory effects of thyrotropin-releasing hormone (TRH) may be mediated by TSH. In rats, the increase of splenocyte proliferative response after TRH administration *in vivo* was abolished by anti-TSH antibodies (26). Most importantly, multiple cell populations of hematopoietic lineage, including lymphocytes and DCs, were shown to produce TSH either spontaneously or in response to proinflammatory stimuli or TRH (27–29).

In our study we demonstrated for the first time the influence of systemic rhTSH administration on the structure of peripheral blood immune cells *in vivo*, encompassing selective increase of the percentage of NKT cells and B lymphocytes in athyroid patients. Little is known about the possible TSH–NKT cells’ interactions. The available data pertaining to the influence of thyreometabolic state on NKT consist of an evaluation of the number of NKT cells in 11 patients with Graves’ disease (GD). In those patients no link was found between thyreotoxicosis (low TSH, high HTs) and the amount of NKT cells in the peripheral blood (30). However, in an aforementioned study the influence of TH level fluctuations cannot be excluded and separated from the direct effects of TSH. To the contrary, our results were obtained in a large group of prospectively assessed athyroid patients, allowing analysis of TSH activity on immune system. Although the observed quantitative changes in NKT cells and B lymphocyte populations may still be dependent on the indirect, cumulative effect of other humoral, cellular, or hormonal factors, direct effects of acute rhTSH administration are more plausible in such clinical model.

As indicated earlier, more is known about TSHR expression on particular immune cell subsets in lymphoid organs of laboratory animals (10). Till now, TSHR expression has not been studied in NKT cells, but it was documented on particular subsets of B lymphocytes (10). Interestingly, a study performed with an animal model revealed the impact of hypothalamus pituitary thyroid axis on the frequency and absolute number of pro-B, pre-B cells, and B lymphocytes in the bone marrow of hypothyroid mice (31). The (*hyt/hyt*) hypothyroid strain of mice is characterized by reduced serum levels of THs and up to 100-fold elevated serum levels of TSH (32). In these mice the percentage of B lymphocytes and their precursors was significantly reduced, as compared to normal mice, and administration of THs resulted in a specific increase in the frequency and total number of pro-B cells in peripheral circulation (31). Although in our study the population of peripheral blood B lymphocytes increased after rhTSH administration in patients with stable TH levels, both observations underline the importance of thyrometabolic parameters in the lymphocyte biology. The differences may be dependent on species characteristics and significantly diverse experimental conditions—*i.e.* lack of changes in TH levels in our model and longitudinal *versus* short term elevation of serum TSH concentration as well as other sources of investigated immune cells. Complex and dependent on microenvironment, interactions between particular cell subsets as a result of TSH stimulation should be also taken into consideration. Invariant subset of NKT cells is known to take part in the regulation of the B lymphocyte function on multiple levels, especially in the context of autoimmune and anti-tumor reaction (33, 34).

Additionally, the results of our earlier studies suggest that the influence of thyreometabolic status on the immune system may be modulated by factors associated with the ongoing pathological processes *e.g.* in the thyroid gland. Levothyroxine (L-T4) supplementation in patients after thyroidectomy because of DTC was associated with a significant increase in peripheral blood pDCs and cDCs and an increase in surface expression of CD86 on both DC subpopulations (35). It was also demonstrated that THs increased the ability of human peripheral blood DCs to stimulate the proliferation and secretion of IL-12 by PBMCs *in vitro* (35). However, the effect of L-T4 supplementation was different in patients with Hashimoto’s disease. In this group of patients, the level of pDCs in the PBMC fraction decreased during the L-T4 treatment, while the percentage of cDCs remained unchanged (36).

In our previous work (12) as well as in the current study—performed on a significantly enlarged group of patients, we did not observe any significant influence of systemic administration of rhTSH on the quantity and structure of human peripheral blood DC subpopulations nor on the expression of costimulatory molecule CD86—one of the main DC maturation and activation markers.

Although the expression of TSHR is significantly higher in DCs than in other immune cells from murine lymph node and spleen (10), little is known about the expression of TSHR in human peripheral blood DCs, and this area deserves definitely further research also in the light of our results. Beside the species-specific characteristics, also the difference in the source of examined cells may help in understanding the lack of TSHR effects on DCs in our study. It has been demonstrated that peripheral blood DCs represent mostly immature cells, and they change their phenotype and functional properties upon entering target tissues and organs [reviewed in (37)]. Furthermore, the microenvironmental factors influencing the process of DC activation and maturation may differ considerably between particular localizations. In our earlier analysis we observed *e.g.* differences in DC subsets between peripheral and portal blood are dependent additionally on the pancreatic pathology (38). Thus, it is possible that the high expression of TSHR on DCs in murine lymphatic organs does not reflect the situation in peripheral blood. Additionally, it is highly possible that the TSHR ligation without other inflammatory stimuli is not sufficient for DC activation.

Previous studies demonstrated that biotinylated TSH can bind to human peripheral blood CD14^+^ monocytes (25). It was also shown that in athyroid patients rhTSH administration increased the pro-inflammatory gene expression in monocytes isolated from peripheral blood leukocytes (39). Yet, in our experimental settings, administration of rhTSH in high doses did not impact the percentage of the investigated monocyte subpopulations. The current observation together with the results of the few earlier studies suggests that the influence of TSH on monocytes may be associated with functional but not overt quantitative changes in the main monocyte subpopulations.

The presented findings indicate that rhTSH may have a direct, THs independent, impact on particular populations of peripheral blood immune cells in humans. Still, the role of indirect effects associated with changes of the complex humoral and cellular networks cannot be excluded. Interestingly, in one of the studies rhTSH was suggested as a potential trigger of autoimmune response in GD (40). However, in the large group of patients with DTC remaining in a long-term follow-up in our Department, Graves’ orbitopathy has never occurred. Nevertheless, further investigations seem to be mandatory to resolve the exact short- and long-term effects exerted by rhTSH administration on human immune system. Beside the importance of this observation in the basic knowledge of immunoregulatory mechanisms, better understanding of the immune properties of high systemic levels of TSH can be of special meaning for patients with inflammatory and malignant diseases of the thyroid.

## Data Availability Statement

The raw data supporting the conclusions of this article will be made available by the authors, without undue reservation.

## Ethics Statement

The studies involving human participants were reviewed and approved by the Local Ethics Committee, the Polish Mother’s Memorial Hospital—Research Institute, Lodz, Poland. The patients/participants provided their written informed consent to participate in this study.

## Author Contributions

The conceptualization of the study was done by ZA and MS. The data curation was made by PŚ, KW-S, ZA, and AZ. The formal analysis was done by MS, ZA, PŚ, and AL. The investigation was done by ZA, MS, and PŚ. The methodology was made by MS, PŚ, KW-S, and AL. MS, ZA, and AL were in charge of the project administration. MS was in charge of the resources. MS and AL supervised the study. MS and PŚ validated the study. The visualization was done by PŚ. MS, PŚ, and KW-S wrote the original draft. MS, ZA, AZ, and AL wrote, reviewed, and edited the manuscript. All authors contributed to the article and approved the submitted version.

## Funding

This study was supported by statutory funds from the Medical University of Lodz, Poland (no. 503/1-107-03/503-11-001-19-00).

## Conflict of Interest

The authors declare that the research was conducted in the absence of any commercial or financial relationships that could be construed as a potential conflict of interest.

## Supplementary Material

Supplementary Figure 1 The influence of rhTSH on distribution of human CD16^+^ cells. The percentage of CD16^+^ **(A)**, CD14^−^CD16^+^ **(B)** and CD14^+^CD16^+^ **(C)** cells in the whole PBMC fraction of patients (n = 109) directly before (pre) and on the fifth day of the study (post).

Supplementary Figure 2 The influence of rhTSH on CD86 expression in peripheral blood DC subpopulations. The percentage of CD86 positive cells in **(A)** pDCs and **(B)** CD1c/BDCA1^+^ cDCs in the DC fraction of patients (n = 109) directly before (pre) and on the fifth day of the study (post).
